# Permanent Stress Adaptation and Unexpected High Light Tolerance in the Shade-Adapted *Chlamydomonas priscui*

**DOI:** 10.3390/plants13162254

**Published:** 2024-08-14

**Authors:** Devon Popson, Susanna D’Silva, Kaylie Wheeless, Rachael Morgan-Kiss

**Affiliations:** Department of Microbiology, Miami University, Oxford, OH 45056, USA; popsond@miamioh.edu (D.P.); dsilvas@miamioh.edu (S.D.); wheele34@miamioh.edu (K.W.)

**Keywords:** photoinhibition, photo-acclimation, extremophile, cyclic electron flow, environmental change

## Abstract

The Antarctic photopsychrophile, *Chlamydomonas priscui* UWO241, is adapted to extreme environmental conditions, including permanent low temperatures, high salt, and shade. During long-term exposure to this extreme habitat, UWO241 appears to have lost several short-term mechanisms in favor of constitutive protection against environmental stress. This study investigated the physiological and growth responses of UWO241 to high-light conditions, evaluating the impacts of long-term acclimation to high light, low temperature, and high salinity on its ability to manage short-term photoinhibition. We found that UWO241 significantly increased its growth rate and photosynthetic activity at growth irradiances far exceeding native light conditions. Furthermore, UWO241 exhibited robust protection against short-term photoinhibition, particularly in photosystem I. Lastly, pre-acclimation to high light or low temperatures, but not high salinity, enhanced photoinhibition tolerance. These findings extend our understanding of stress tolerance in extremophilic algae. In the past 2 decades, climate change-related increasing glacial stream flow has perturbed long-term stable conditions, which has been associated with lake level rise, the thinning of ice covers, and the expansion of ice-free perimeters, leading to perturbations in light and salinity conditions. Our findings have implications for phytoplankton survival and the response to change scenarios in the light-limited environment of Antarctic ice-covered lakes.

## 1. Introduction

Most photosynthetic organisms encounter marked light fluctuations, ranging from insufficient to excessive, occurring over broad timescales. Short-term fluctuations (e.g., min to h) in irradiance can result in variations by up to one thousand times the daily average irradiance, representing an acute stressor [1]. Under optimal light intensities, photosynthetic organisms balance photosynthetic energy production with metabolic needs; however, light levels that exceed energy consumption rates by downstream metabolism can cause photooxidative damage to components of the photosynthetic electron-transport chain [2,3]. Moreover, light requirements for photosynthesis are significantly altered in the presence of environmental stressors, such as heat, chilling, nutrient deprivation, and salinity [4].

Photooxidative stress generates reactive oxygen molecules (ROS) that can damage various proteins of the photosynthetic electron transport chain, with photosystem II (PSII) being the primary target, leading to a reduction in electron flux capacity [5,6]. Specifically, D1 and D2 reaction center proteins have several amino acid targets that are modified by the ROS hydroxyl radicals (HO) and superoxide anions (O_2_^−^) [7,8]. Accordingly, plants and algae have evolved an efficient repair system to rapidly replace damaged PSII centers [9]. A vast body of knowledge has been generated on PSII photodamage and repair. On the other hand, photosystem I (PSI) is generally considered to be more stable compared to PSII; however, PSI appears to be very sensitive to oxidative damage under specific environmental conditions (e.g., low temperature, fluctuating light, and drought) [6,10,11,12,13]. Thus, PSI damage can contribute to losses in photosynthetic efficiency photochemistry, particularly under stress conditions [14]. For example, several chilling-sensitive crop species exhibit irreversible PSI damage under low temperatures/mediate light, a typical condition during winter and spring. PSI damage is also fundamentally different from that of PSII: it involves the destruction of the iron–sulfur centers, followed by the destruction of other electron acceptors [15]. Therefore, there is not an efficient PSI repair cycle, and PSI photoinhibition results in slower recovery and higher energy costs [16].

Efficient photosynthesis requires photostasis, or a balance between light absorption and turnover of ATP and NADPH via metabolic processes. Plants and algae have evolved numerous strategies to mediate potential imbalances in light absorption and utilization during exposure to high light intensities, fluctuating light, or additional environmental stress conditions that influence light utilization. These mechanisms include reduced light harvesting, state transitions, non-photochemical quenching (NPQ), and PSI cyclic electron flow (CEF) [2,11,17,18]. Collectively known as photoprotective mechanisms, these strategies may be induced across different timescales to mediate damage or rebalance energy production to energy needs.

While excess light is a stressor for all photosynthetic organisms, the intensity of light that induces stress is highly variable between organisms and is largely dependent on the native light environment [19]. Additionally, pre-acclimation to certain environmental conditions can impact an organism’s tolerance and response to photoinhibition [6,20,21,22,23]. For example, the combination of low temperatures and high-light stress can cause enhanced photoinhibition of PSII and PSI; however, pre-acclimation to low temperatures prior to short-term high-light exposure confers increased resistance to high-light stress [6,21]. This resistance to photoinhibition can be further enhanced by acclimation to low temperature and high light [22]. On the other hand, the acclimation to some stressors appears to exacerbate high light sensitivity: high salt stress can increase photoinhibition by impairing the D1 repair cycle [20,23]. While extensive research has been conducted on model organisms to understand the impact of light stress, there is growing interest in how organisms with high tolerance to permanent environmental stressors mediate their stress response [24]. Exploring the physiological diversity of a larger range of photosynthetic organisms may uncover novel approaches for improving stress resistance in crop species. Thus, work characterizing these “non-model organisms” and their response to photoinhibition could provide greater insights into how robust photosynthesis can be maintained in suboptimal or even extreme conditions.

*Chlamydomonas priscui* (formerly *Chlamydomonas* sp. UWO241; hereafter UWO241) is an extremophilic alga isolated from Lake Bonney in the McMurdo Dry Valleys, Antarctica. Lake Bonney has a permanent ice cover (3–5 m thick) that results in a perennially stratified water column characterized by steep gradients in nutrient and ion chemistry, temperature, and light [25]. Within this unique habitat, UWO241 has been isolated under permanent low temperatures, high salt, and extreme shade [4]. Both low temperatures and high salt stress induce oxidative stress; conversely, low light is associated with lower oxidative stress but also limits photosynthetic activity [26]. Moreover, Lake Bonney is experiencing climate-related change, driven by summer glacial melt, which could lead to complex changes in the light, nutrients, and salinity environment experienced by the phytoplankton communities.

For over three decades, UWO241 has been the subject of intensive research interest due to its unique structural and functional features within its photochemical apparatus, which enables its survival in such an extreme environment [4,27,28]. Specifically, UWO241 exhibits constitutively high rates of CEF, which is supported by the formation of a PSI-Cyt b_6_f supercomplex and can support increased ATP production under salt stress [29,30]. UWO241 also lacks the capacity for the short-term mechanism of state transitions despite the presence of Stt7 and Stl1 kinases [31,32]. Recently, Stahl-Rommel et al. reported that NPQ, CEF, and the ascorbate glutathione cycle contribute to the ability of UWO241 to grow under low temperatures, high salt, and high light [33]. These findings are also supported by previous reports that UWO241 employs CEF and NPQ as long-term protective strategies [4,30,34]. The reliance on CEF for long-term stress acclimation is distinct from other model algae and higher plants, which typically activate these mechanisms in response to short-term high-light stress [35,36]. Overall, research so far suggests that UWO241 has forgone a broader range of photoprotective mechanisms in favor of the increased capacity of a few key mechanisms. While photoprotective mechanisms that are constitutively “on” are a benefit under a climate regime favoring extreme, stable conditions of Lake Bonney, reduced physiological flexibility could limit the organism’s ability to respond to future scenarios of environmental variation.

UWO241 represents a key phytoplankton taxon in the dry valley lake food web, as well as a resource for understanding stress-adapted photosynthesis. The majority of previous literature on UWO241 has focused on the implications of its tolerance for low temperatures [32,34,37,38] or high salinity [30,39]. Climate change-associated thinning ice covers and an increasing ice-free lake perimeter will shift the light environment to higher and more variable summer irradiance regimes [40]. Moreover, these changes in the light environment will be associated with variability in other conditions, including temperature and salinity, as a result of increased water column mixing. In recent years, green algae have been shown to be increasing in abundance in Lake Bonney, suggesting they may play an increasingly important role in this ecosystem as the effects of climate change continue [41]. Here, we evaluated the implications of long-term high light acclimation on the growth and photochemical function of UWO241, as well as the impact of adaptation and acclimation to low temperatures, high salt, or high light on the short-term high-light stress response. Notably, we found that UWO241 shows an increased growth rate and concomitant increased O_2_ evolution under high-light conditions. We also showed that not only does UWO241 retain the ability to mediate short-term high-light stress, but it is highly resistant to PSI photoinhibition. Additionally, acclimation to high-growth light and low temperatures confer increased resistance to PSII photoinhibition during short-term high-light stress, whereas high salt increases photoinhibition. Thus, the adaptation of UWO241 to extreme conditions has implications on its ability to tolerate short-term photoinhibition as well as survival under climate-driven variability in temperature and salinity.

## 2. Results

### 2.1. Growth Patterns under High-Light Conditions

**Figure 1:**
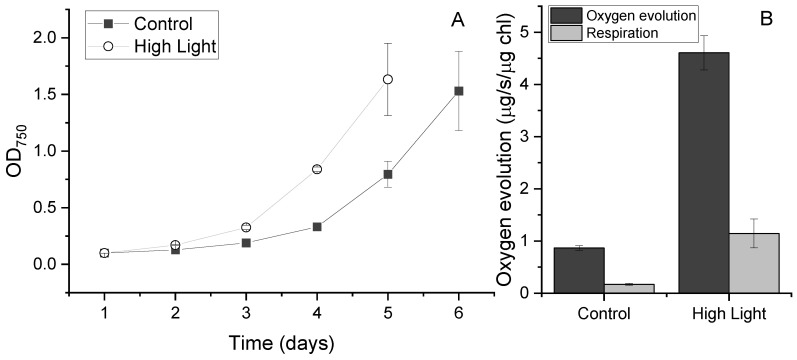
Growth (**A**) and oxygen evolution under saturating light (**B**) in *C. priscuii* UWO241 under low and high light. Cultures were acclimated and grown under control (50 μmol m^−2^ s^−1^) or high-light (250 μmol m^−2^ s^−1^) conditions. (*n* = 3; ±SD).

To determine how increased light availability would impact the growth kinetics of UWO241, we monitored its growth under control (50 μmol m^−2^ s^−1^) and high-light conditions (250 μmol m^−2^ s^−1^). These light conditions were chosen to represent typical native light conditions under the ice versus light levels in the ice-free, open-water perimeter moats that form at the perimeters of the Antarctic lakes during the late summer, respectively (Figure S1) [42]. When compared with low-light growth conditions, UWO241 grown under high-light conditions exhibited a lag phase of one day shorter and a 1.2-fold increase in growth rate (Figure 1A, Table 1). While the high-light-grown cultures also had a lower total chlorophyll concentration (Table 1, *p* < 0.0005), there was no significant change in the chlorophyll a/b ratio (chl a:b) between high-light vs. control cultures (Table 1, *p* = 0.25). The maximum photochemical efficiency (F_V_/F_M_) was reduced by 1.40-fold in the high-light versus control cultures.

Photosynthetic activity was probed with oxygen evolution and photosynthetic irradiance curves. Under high light, there was a 4.5-fold increase in the maximum rate of oxygen evolution relative to that of the control (Figure 1B, *p* < 0.0005). A similar trend was seen in the respiration rate, with a 6.0-fold increase in rates seen in the response to the higher-growth light (Figure 1B, *p* < 0.0005).

**Figure 2:**
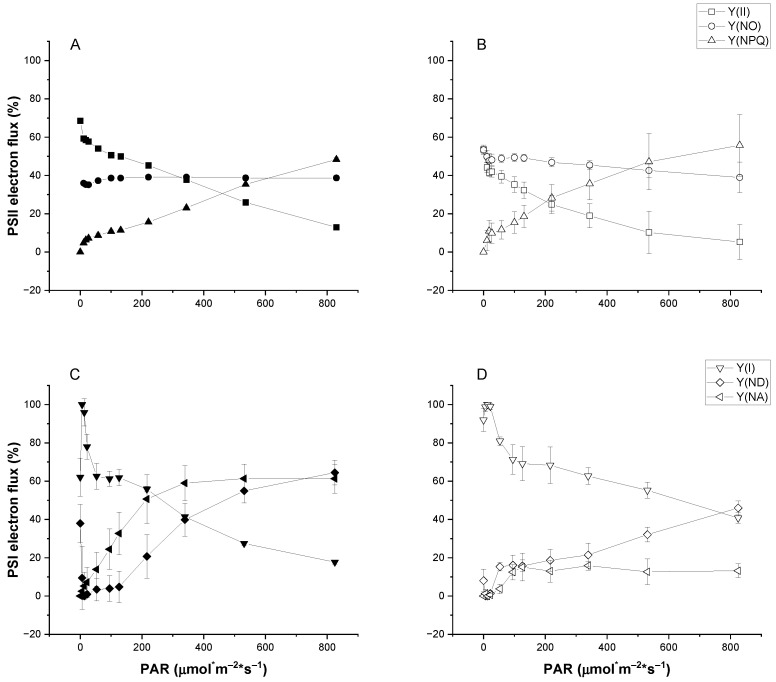
Rapid light curves for PSII (**A**,**B**) and PSI (**C**,**D**) quantum yields of UWO241 acclimated to control (closed symbols) or high light (open symbols). Y(II) and Y(I), quantum yields for PSII and PSI, respectively. Y(NPQ) and Y(NO), regulated and unregulated nonphotochemical quenching of PSII, respectively; Y(ND) and Y(NA), energy loss due to donor- and acceptor-side limitations, respectively (*n* = 3; ±SD).

Rapid light curves revealed distinct saturation characteristics for both PSII and PSI in high light-versus control-grown cultures of UWO241. Compared to control conditions, high-light-grown cultures exhibited moderately lower values of [Y(II)], which reflected a reduction in the energy fraction used for PSII photochemistry. This trend was coupled with an increase in the quantum yield of regulated energy dissipation, Y(NPQ). On the other hand, non-regulated energy dissipation, [Y(NO)], remained relatively constant across all light intensities (Figure 2A,B).

The quantum yield of PSI [Y(I)] revealed that control-grown cultures exhibited higher PSI sensitivity to increasing light intensities. Under excess light, the minimum value for Y(I) was lower in control (18%) vs. high light-grown (26%) cultures. Lower Y(I) values in the control cultures were associated with both donor- and acceptor-side limitation [Y(ND) and Y(NA), respectively], with both parameters reaching 60% at excess light levels (Figure 2C,D). In contrast, high-light-grown cultures exhibited a maximum Y(ND) of 40% and minimal changes in Y(NA) over the light curves (Figure 2D).

### 2.2. Impact of Short-Term High Light on PSI and PSII

**Figure 3:**
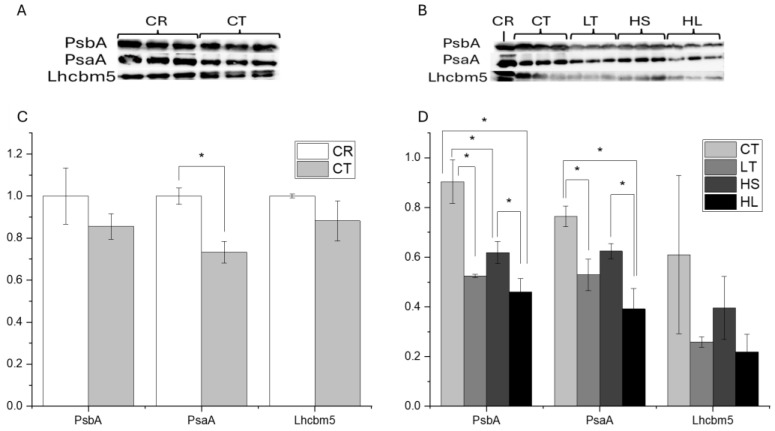
Relative abundance of key photosynthetic proteins in *C. reinhardtii* versus UWO241 (**A**) and in UWO241 cultures acclimated to control, low-temperature, high-salt, or high-light conditions (**B**). Quantification of relative protein abundances via densitometry are shown for *C. reinhardtii* vs UWO241 (**C**) and between stress acclimated condition of UWO241 (**D**). Thylakoid samples were normalized to 6 µg of protein and run in triplicate. Densitometry was performed in ImageJ and expressed relative to *C. reinhardtii*. * Indicates *p* < 0.05 based on a Welch’s *t*-test (panel A) or ANOVA with Tukey post hoc test (panel B) (*n* = 3; ±SD).

Next, we probed the consequences of short-term high-light stress, or photoinhibition, on both PSII and PSI in combination with long-term stress factors, namely, low temperature (LT), high light (HL), or high salt (HS). Prior to the photoinhibition assays, we compared the abundances of three major photosynthetic proteins between UWO241 grown under non-stressed/control conditions (CT) and C. *reinhardtii* (CR). The relative abundance of PSI and PSII core proteins (PsaA and PsbA) and light-harvesting complex (Lhcbm5) for all conditions were compared prior to the short-term stress (Figure 3A). Western blots showed that CT UWO241 cells had comparable levels of the PSII core and light-harvesting protein, PsbA and Lhcbm5, respectively, as *C. reinhardtii*; however, UWO241 exhibited 1.3-fold lower levels of the PSI core protein, PsaA compared with control-grown cells of *C. reinhardtii* (Figure 3C; *p* = 0.002).

The abundance of PsbA, PsaA, and Lhcbm5 was also evaluated in UWO241 cultures grown under control conditions versus long-term stress (low temperature, high salt, high light) (Figure 3B). Relative to control conditions, the abundance of all three proteins was lower in UWO241 when acclimated to all three stress conditions; however, only low temperature or high salt was significantly different from the control (*p* < 0.001, *p* = 0.034, respectively).

**Figure 4:**
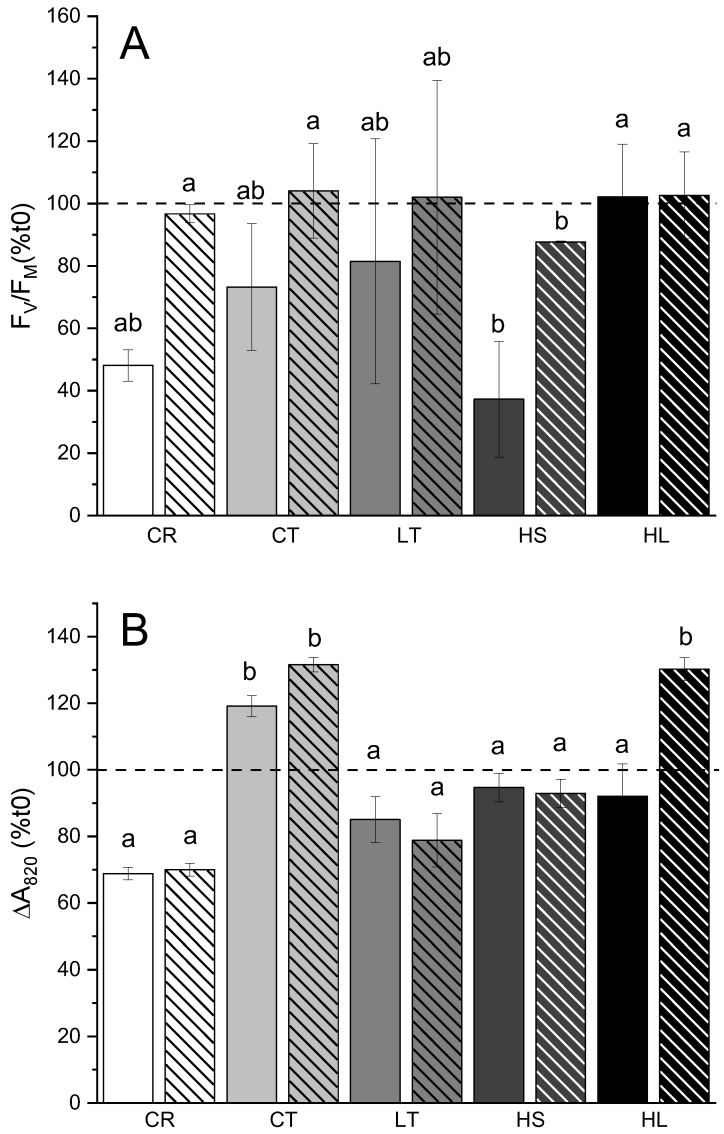
Changes in photosynthetic capacity following short-term high-light stress (120 min, 8 °C/1000 μmol m^−2^ s^−1^; solid bars) and recovery in (90 min growth conditions; striped bars) (**A**) PSII photoinhibition shown by changes in F_V_/F_M_ (*n* = 3; ±SD); (**B**) PSI photoinhibition represented as change in absorbance at 820 nm, relative to pre-stress time point, t_0_ (*n* = 9; ±SE). Statistical differences were calculated via two-way ANOVA with Tukey’s post hoc test (*p* ≤ 0.05). Solid line separates *C. reinhardtii* (left bars) from UWO241 (right bars). Dotted line represents the relative value (100%) for each strain or condition taken immediately prior to the photoinhibition treatment. Strain and growth conditions: CR, *C. reinhardtii*; CT, UWO241, control; LT, UWO241, low temperature; HS, UWO241, high salt; HL, UWO241, high light.

PSII and PSI photoinhibition were monitored in *C. reinhardtii* and UWO241 under control and stressed conditions. *C. reinhardtii* and UWO241 grown under control conditions exhibited similar trends in high-light-induced 31 and 52% loss in PSII capacity, respectively, followed by full recovery (Figure 4A). Pre-acclimation of UWO241 to either low temperature or high light eliminated the photoinhibition-induced reduction in F_V_/F_M_. However, high-salt-acclimated cells exhibited increased sensitivity of PSII capacity and failed to fully recover following photoinhibition treatment (Figure 4).

In contrast with PSII, *C. reinhardtii* exhibited higher sensitivity of PSI in response to photoinhibition compared to UWO241. *C. reinhardtii* exhibited a 30% reduction in PSI capacity, which did not recover (Figure 4B). In contrast, control-grown UWO241 exhibited a 20% increase in PSI capacity in response to the photoinhibition treatment, which was maintained through the recovery period. Pre-acclimation to stress conditions generally did not affect the PSI response of UWO241. One exception was that, similar to control-grown cells, high-light-grown cultures also showed an increase in PSI activity during the recovery period (*p* = 0.003).

**Figure 5:**
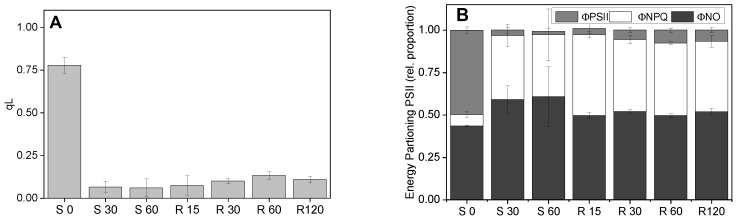
Impact of photoinhibition on PSII oxidation state (**A**) and energy partitioning (**B**). Cultures of UWO241 were exposed to high light for 60 min and then recovered in low light for 120 min. S0, S30, and S60: high light exposure for 0, 30, and 60 min; R15, R30, R60, and R120: recovery under growth conditions for 15, 30, 60, and 120 min (*n* = 3; ±SD).

While the results of Figure 4 show that UWO241 has a robust response to short-term high light at the level of both PSII and PSI capacity, other PSII parameters exhibited high sensitivity to high-light stress (Figure 5). The PSII redox state (qL) and quantum yield of PSII (ΦPSII) exhibited significant reductions after only 30 min of exposure to 300 μmol m^−2^ s^−1^ (Figure 5). Loss in ΦPSII was accompanied by an increase in ΦNPQ. Moreover, full recovery of F_V_/F_M_ after exposure to short-term high light (Figure 4A) and no relaxation of qL was observed after 2 h of recovery, suggesting that the PQ pool remained over-reduced after short-term high light treatment. This result was also accompanied by the persistence of low ΦPSII, indicating PSII that remained downregulated. Similarly, ΦNPQ remained relatively high (Figure 5B).

**Figure 6:**
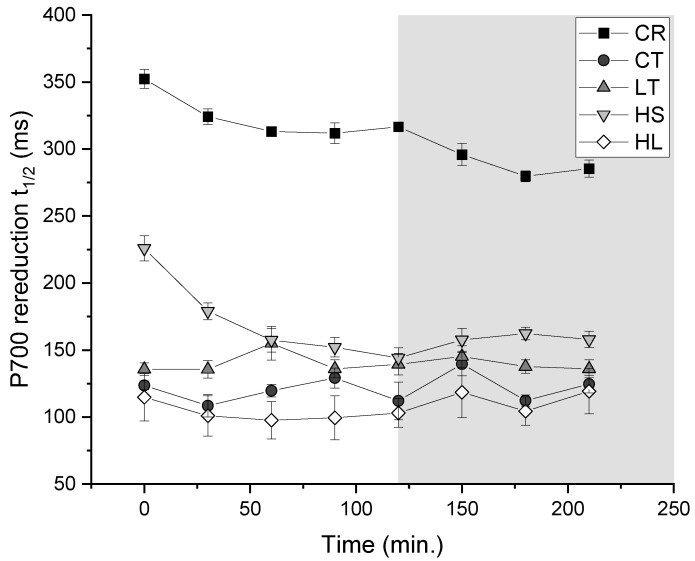
Rates of CEF measured as the half-time of P700 re-reduction throughout the short-term stress (1000 μmol m^−2^ s^−1^/8 °C; white) and subsequent recovery at the respective growth conditions (grey). Strain and growth conditions: CR, *C. reinhardtii*; CT, UWO241, control; LT, UWO241, low temperature; HS, UWO241, high salt; HL, UWO241, high light (*n* = 3; ±SE).

Re-reduction rates of P700 were monitored as an estimate of PSI-driven cyclic electron flow (i.e., CEF) in response to photoinhibition and exhibited strain- and treatment-specific trends. Overall, UWO241 exhibited significantly faster P700 re-reduction rates, i.e., higher CEF, compared with *C. reinhardtii.* In response to photoinhibitory treatment, control-grown *C. reinhardtii* responded with a modest (0.23-fold) increase in CEF (Figure 6). All cultures of UWO241 maintained relatively high CEF throughout the treatment. High-salt-grown UWO241 initially had lower rates of CEF than any other growth condition but reached comparable rates after 30 min of stress.

### 2.3. ROS Levels and Response to Chemical Oxidants

**Figure 7:**
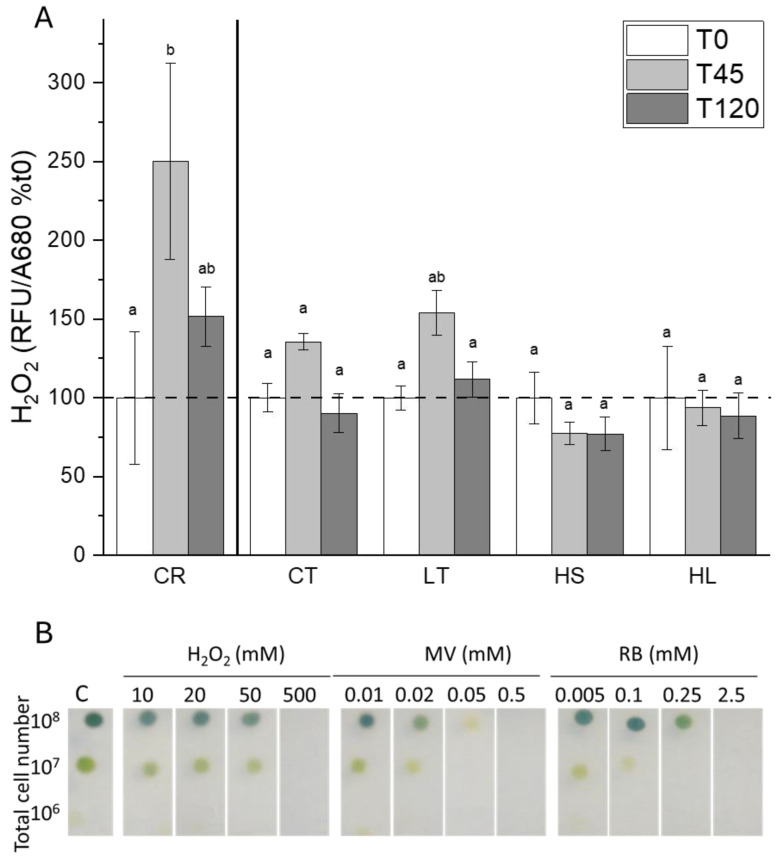
(**A**) Accumulation of H_2_O_2_ in response to the short-term high-light stress as measured by fluorescence of H_2_DCFDA (*n* = 9; ±SE). Statistical differences are based on a two-way ANOVA with Tukey’s post hoc test (*p* ≤ 0.05). (**B**) Sensitivity of UWO241 to multiple reactive oxygen species. Cultures were spotted on agar plates containing varying concentrations of chemical oxidants (Rose Bengal and Methyl Viologen produce singlet oxygen and superoxide, respectively). Strain and growth conditions: CR, *C. reinhardtii*; CT, UWO241, control; LT, UWO241, low temperature; HS, UWO241, high salt; HL, UWO241, high light.

A recent paper reported on the capacity of UWO241 for maintaining low ROS levels through relatively high activity of the glutathione–ascorbate pathway [33]. We monitored the levels of H_2_O_2_ as a proxy for ROS during short-term photoinhibition. While both strains exhibited an increase in H_2_O_2_ in response to high-light stress, *C. reinhardtii* showed a significantly higher rise in ROS. UWO241 cultures grown under either HS or HL exhibited no significant change in ROS (Figure 7A).

Since UWO241 exhibited a relatively high capacity to maintain low ROS (Figure 7A), sensitivity to exogenously supplied ROS was tested in the presence of chemical oxidants using an agar plate assay (Figure 7B). UWO241 sensitivity was tested in the presence of H_2_O_2_, singlet oxygen (Rose Bengal), and superoxide (Methyl Viologen). UWO241 exhibited resistance to exogenous H_2_O_2_ in all tested concentrations <500 mM H_2_O_2_. On the other hand, cells exhibited higher sensitivity to both Rose Bengal and Methyl Viologen (Figure 7B).

## 3. Discussion

Early literature often referred to *C. priscui* UWO241 as an extremophilic alga adapted to “extreme shade” [43,44]. There was also evidence of a lack of physiological plasticity in the alga, including a requirement for low temperatures, an inability to grow in red light [45], and loss of a classic short-term acclimation mechanism, state transitions [31]. Our findings support earlier reports that UWO241 can grow at light levels far greater than native conditions under permanent ice [4]. In addition, our study agrees with that of Pocock et al., whereby UWO241 possesses some capacity for tolerating short-term excessive light levels [45]; however, the response to photoinhibition of PSII is nuanced.

UWO241 exhibited a modest increase in growth rate under high (250 μmol m^−2^ s^−1^) relative to control (50 μmol m^−2^ s^−1^) irradiance conditions. On the other hand, the rates of O_2_ evolution increased substantially on a per-chl basis (400%) in high-light-grown cultures. Normalizing to cell counts results in a similar rate of O_2_ evolution between high-light and control cultures (Figure S2), indicating that the high-light cultures are more efficient photosynthetically, achieving a higher rate of O_2_ evolution per unit of chl. It is also likely that other processes contributed to the high O_2_ production, such as the water–water cycle or chlororespiration. Stahl-Rommel et al. reported that UWO241 has a highly active ascorbate cycle [33]. In addition, HL-grown cultures showed evidence of downregulation of PSII: F_V_/F_M_ was lower, and Y(II) declined faster in the rapid light curves (Table 1; Figure 2). UWO241 shows no change in chl a:b ratio in response to high-growth light but rather decreases total chlorophyll levels, ultimately decreasing the number of reaction centers rather than adjusting antennae size to regulate the light-harvesting capacity. This is supported by Western blot analysis of PsbA and PsaA, which showed lower concentrations of all proteins in the high-light-acclimated cultures relative to control conditions (Figure 3). Typically, adjusting the light-harvesting capacity via antennae size regulation is seen as a hallmark component of long-term, high-light acclimation [46]. However, our results agree with previous studies that UWO241 maintains a consistently low chl a:b ratio under various stressors, including differing light quality, high salinity, and low temperature [33,47].

UWO241 relies mainly on constitutive protection strategies during short-term high-light stress. PSII photoinhibition of the control-grown cultures showed comparable rates of photoinhibition and recovery of F_V_/F_M_ to *C. reinhardtii*. On the other hand, Y(II) remained low during the recovery period after photoinhibition (Figure 5). This suggests that while primary PSII photochemistry may have recovered, there is a nuanced response to photoinhibition within the photosynthetic electron transport chain. Persistent NPQ suggests that UWO241 maintained a long-term energy-dissipation capacity in the absence of high-light stress. This is supported by the inability of qL to relax, suggesting ongoing issues with the electron transport chain or downstream metabolism. It is also notable that CEF rates did not relax during the recovery phase in any of the treatments (Figure 6). Together, these results indicate that while UWO241 may have the capacity to protect PSII photochemistry, the photosynthetic electron transport chain is very slow to recover from short-term high light. This has implications for future ice-free scenarios in the Antarctic lakes.

Pre-acclimated UWO241 to some stressors conferred additional photoprotection under short-term high-light stress. Growth under low temperature or high light resulted in full protection of PSII photochemistry (i.e., F_V_/F_M_) to photoinhibition. These data suggest that, similar to other model species, long-term acclimation enhances high-light tolerance in UWO241. In contrast, cells pre-acclimated to high salt exhibited higher sensitivity to photoinhibition and incomplete recovery of F_V_/F_M_. Our high-salt condition matches the native salt levels within the deep photic zone of Lake Bonney, where light levels are extremely low. Pocock suggested that a rapid D1 repair cycle is critical to the photoinhibition response of UWO241 [48]. In this study, we also observed the rapid recovery of PSII capacity apart from the high-salt condition. High salt stress has been shown to impair the D1-repair cycle [20]. This suggests that elevated PSII photoinhibition seen in high-salt-acclimated cultures could be due to a lack of repair rather than increased rates of PSII photodamage. Thus, a combined high-light/high-salt condition within its native habitat represents an additional stressful environment that UWO241 is not well equipped to deal with.

PSI function appears to be especially robust in UWO241. First, HL-grown UWO241 exhibited an increased capacity for avoiding donor- or acceptor-side limitation (Figure 2). In addition, unlike *C. reinhardtii*, UWO241 exhibited little to no PSI photoinhibition despite the combined low-temperature and high-light conditions. While PSII is the primary target of photoinhibition, combined low-temperature and high-light stress have been shown to induce PSI photoinhibition in cold-sensitive and tolerant plants [6,21]. However, acclimation to high excitation pressure, such as the low temperatures that UWO241 experiences in its native environment, can prime against PSI photoinhibition [21]. The pre-acclimation of UWO241 to low temperatures, high light, or salt did not impact PSI activity in response to the short-term light stress. A robust PSI would be an asset in its native habitat, where environmental conditions such as low temperatures and high salinity would put PSI at a high risk of oxidative damage.

UWO241 exhibits two potential strategies in its photoinhibition response that could contribute to PSI photoprotection: both involve tight regulation of ROS production and/or detoxification. While *C. reinhardtii* showed a rise in H_2_O_2_ during exposure to short-term light stress, under all conditions, UWO241 maintained very low H_2_O_2_ in response to the stress. H_2_O_2_ is produced in the thylakoid membrane via disproportionation of O_2_^−^ at PSI [49,50]. This H_2_O_2_ can then be reduced to H_2_O via the ascorbate glutathione cycle. Accordingly, UWO241 has been shown to exhibit a highly active ascorbate glutathione cycle [33], which likely facilitates this ROS control.

CEF may also play a role in its resistance to PSI photoinhibition by alleviating both donor- and acceptor-side limitation, and preventing ROS production. UWO241 exhibits constitutively high rates of CEF under low temperatures, high salt, and high light (Figure 4; [4,30,33]). In contrast with *C. reinhardtii*, CEF was not stimulated in response to short-term high-light stress in UWO241, with the exception of cultures acclimated to low temperatures. Under stress, CEF can help to alleviate PSI acceptor-side limitations while also contributing to proton motive force to drive NPQ or increase ATP production [51,52,53]. Recent studies in plants have shown that under both low-temperature and high-light stress, CEF tends to support photoprotection rather than energy generation [54,55]. Alternatively, UWO241 utilizes high CEF for ATP production under high-salt stress [30]. This differential functioning of CEF could also contribute to the opposing influences of preacclimation to low temperature and high light versus high salt.

Our study extends the understanding of the advantages and tradeoffs of adaptation to permanent, extreme stress experienced by the photopsychrophile UWO241. UWO241 exhibits tolerance to both short- and long-term light levels that far exceed its native habitat light environment. These results suggest that the organism has some ability to respond to and survive climate-related changes to its light environment, including thinning ice and even ice-free conditions. While there is evidence of photoprotection of PSII and PSI photochemistry, high light also has a significant impact on additional photosynthetic electron-transport processes, the implications of which are not yet fully understood. Furthermore, other stressors, such as high salt, are modulators of this organism’s capacity for light tolerance.

## 4. Materials and Methods

### 4.1. Growth and Stress Conditions

*Chlamydomonas priscui* (CCMP1619; formerly called *Chlamydomonas* sp. UWO241) and *Chlamydomonas reinhardtii* (UTEX89) cultures were grown in Bold’s Basal Media (BBM) in 250 mL glass Pyrex tubes, which were suspended in temperature-controlled aquaria, with filtered air bubbled into the tubes by aquarium pumps with a 24 h light cycle [56]. The light, temperature, and salt regimes are outlined in Table 2. The high light represents the maximum light intensity experienced in the Lake Bonney water column (Figure S1) and the maximum irradiance under which UWO241 can grow [4]. Three biological replicates of each condition were grown for all experiments conducted. Culture density was measured at a wavelength of 750 nm (OD_750_) using a spectrophotometer (UV-1700 PharmaSpec, Shimadzu, Kyoto, JP), and cell counts were monitored using an automated cell counter (Countess 3 FL, ThermoFisher, Waltham, MA, USA).

For the short-term stress experiments, cultures were grown to mid-log phase determined by an OD_750_ between 0.7 and 1. The tubes were then transferred to a tank with temperature-controlled aquaria with a temperature/light treatment of 8 °C/1000 μmol m^−2^ s^−1^ for 120 min to induce light stress. Light was supplied by full spectrum LED lights. After treatment, cultures were returned to growth conditions to recover for 90 min.

To monitor the recovery of PSII photochemistry, mid-log phase cultures of UWO241 were exposed to short-term stress (300 µmol m^−2^ s^−1^) for 60 min, then allowed to recover from the stress for 2 h. PSII redox state (qL) and energy partitioning (ΦPSII, ΦNPQ, and ΦNO) were measured at regular time intervals using Chl a fluorescence.

### 4.2. Oxygen Evolution

Oxygen evolution was measured using the Clark-type oxygen electrode (Hansatech Instruments Ltd., Kings Lynn, UK). Then, 2 mL of culture was dark-adapted on ice for 5 min on ice with 10 mM of NaHCO_3_. Oxygen evolution was monitored for 2–3 min under saturating light conditions. The light was then turned off to measure respiration rates. All measurements were performed at 8 °C. The rate of oxygen evolution and respiration were calculated using a linear fit of 1 min of activity and standardized to chlorophyll content.

### 4.3. Chlorophyll Determination

Chlorophyll was extracted in 90% *v*/*v* acetone. Cells were broken via beadbeating (Biospec Inc., Bartlesville, OK, USA) with zirconia/silica beads (0.1 mm) in two 45 s cycles. Absorbance was measured at 647 nm and 664 nm. Total chlorophyll and chlorophyll a and b concentrations were calculated as described in [57].

### 4.4. Western Blotting

Mid-log cultures were collected and spun down at 3200× *g* for 10 min. Thylakoids were isolated as described in [56]. All buffers contained 1 mM of benzamidine and 1 mM of caproic acid, which was added immediately prior to use. Isolated thylakoids were flash-frozen and stored at −80 °C until use.

SDS-PAGE was performed using the Bio-Rad Mini-Protean system. Samples were loaded on an equal 6 µg protein basis and run on a 12% (*w*/*v*) stacking gel containing 6 M of urea and 5% (*w*/*v*) stacking gel as described in [31]. The SDS_PAGE gels were transferred electrophoretically to nitrocellulose membranes on ice for 2 h at 80 V. Membranes were blocked overnight at 5 °C in 20 mM of Tris buffer containing 15 mM of NaCl, 10% (*w*/*v*) powdered milk, and 0.05% (*v*/*v*) Tween 20. The membranes were then probed with PsbA, PsaA, or Lhcbm5 antibodies (Agrisera) followed by an anti-rabbit antibody conjugated to horseradish peroxidase enzyme. A chemiluminescent ECL-HRP system was used, and blots were scanned using a Chemidoc imager (ChemiDoc MP, Biorad, Hercules, CA, USA). Densitometry was performed using ImageJ (v 1.53K) software.

### 4.5. Room Temperature Fluorescence

All fluorescence measurements were performed using a Dual-PAM-100 (Heinz Walz GmbH, Effeltrich, Germany). For PSII fluorescence measurements, 2 mL of liquid culture was dark-adapted for 2 min under far-red light and supplemented with 10 mM of NaHCO_3_. Samples for PSI fluorescence were dark-adapted for 10 min before being filtered onto a 25 mm GF/C filter (Whatman) and placed in the leaf attachment.

Photosynthetic Light Curves measuring PSI and PSII quenching parameters in control or high-light-grown cultures were monitored at increments of PAR from 0 to 830 μmol m^−2^ s^−1^.

PSII capacity was measured as F_V_/F_M_ and monitored using the induction curve setting as described in [34]. All measurements were performed under temperature and light conditions equivalent to growth conditions. F_V_/F_M_ measurements were taken at time 0 and every 10 min for the first 30 min of light stress and then every 30 min for the remainder of the treatment. The same process and time points were repeated throughout the recovery period.

### 4.6. P700 Measurements

P700 absorbance measurements were taken using the leaf attachment of the PAM-fluorometer to determine PSI activity and CEF rate. A total of 10 mL of culture was treated with 10 μM of DCMU to inhibit PSII activity and dark-adapted for 10 min. A volume equivalent to 5 OD_750_ was filtered onto a 25 mm GF/C filter (Whatman). Actinic red light was used to measure absorbance changes at 820 nm, as outlined in [31]. ΔA_820_ was used as a measure of PSI activity. CEF rate was calculated as the re-reduction half time (t1/2) of P700 using Microcal Origin Software 2024 v10.10 (Microcal Software), as described in [21]. PSI measurements were taken every 30 min throughout the treatment and recovery periods.

### 4.7. ROS Determination

Relative H_2_O_2_ concentrations were measured following the method described in [33]. Pelleted cells were resuspended in 10 mM of TRIS-HCl pH = 7.3, broken via beadbeating, and stored at −80 °C. Thawed samples were normalized to 90 µg of protein and incubated with the fluorescent dye H_2_DCFDA for 30 min at 30 °C. Samples were excited at 485 nm, and fluorescence emission was measured at 535 nm using a plate reader (SpectraMax iD5, Molecular Devices, San Jose, CA, USA).

Sensitivity to exogenously supplied chemical oxidants that produce various ROS was determined using an agar plate assay according to [58]. Cultures were grown to mid-log phase and were serially diluted to achieve a range of total cells (105, 106, 107, and 108) and spotted onto BBM plates containing variable concentrations of the pro-oxidants Rose Bengal, Methyl Viologen, or hydrogen peroxide. A range of concentrations of Rose Bengal (0.005 μM, 0.1 μM, 0.25 μM, and 2.5 μM) was used to induce singlet oxygen, while Methyl Viologen (0.01 μM, 0.02 μM, 0.05 μM, and 0.5 μM) was used to induce superoxide. Hydrogen peroxide exposure was 0.01 mM, 0.02 mM, 0.05 mM, and 0.5 mM. Plates were incubated for 7 days under control growth conditions. Colony growth was visually inspected daily to obtain a qualitative estimate of sensitivity to each of the chemical oxidants.

## Figures and Tables

**Table 1:** Comparison of physiological parameters of *C. priscui* UWO241 grown under control (50 μmol m^−2^ s^−1^) or high light (250 μmol m^−2^ s^−1^). Chlorophyll, cell counts, and F_V_/F_M_ measurements were taken during mid-log growth (*n* = 3; ±SD).

	Control	High Light
Growth Rate (h^−1^)	0.019 ± 0.002	0.023 ± 0.001 *
Cell Count (cells/mL)	2.53 × 10^6^ ± 1.22 × 10^6^	1.62 × 10^6^ ± 3.33 × 10^5^
Total Chl. (µg mL^−1^)	8.10 ± 2.50	2.96 ± 2.08 *
Total Chl. (pg/cell)	7.03 ± 2.85	1.05 ± 0.258 *
Chl. a:b	3.65 ± 0.106	4.39 ± 0.758
F_V_/F_M_	0.664 ± 0.008	0.473 ± 0.060 *

* Indicates *p* < 0.05 based on Welch’s *t*-test.

**Table 2:** Growth conditions of *C. reinhardtii* and UWO241.

Strain/Condition	Light Intensity (μmol m^−2^ s^−1^)	Temperature (°C)	Salt Concentration (mM NaCl)
*C. reinhardtii* (CR)	50	20	0.43
UWO241 Control (CT)	50	8	0.43
UWO241 Low Temperature (LT)	50	2	0.43
UWO241 High Salt (HS)	50	8	700
UWO241 High Light (HL)	250	8	0.43

## Data Availability

Data and strains are available upon request from the authors.

## Supplementary Materials

The following supporting information can be downloaded at: https://www.mdpi.com/article/10.3390/plants13162254/s1, Figure S1: Photosynthetically active radiation (PAR) in the Lake Bonney water column; Figure S2: Oxygen evolution per cell of control vs. high light grown UWO241.
