# Supplementary material for: Permanent Stress Adaptation and Unexpected High Light Tolerance in the Shade-Adapted Chlamydomonas priscui
Source: Plants (Basel). 2024 Aug 14;13(16):2254. doi: 10.3390/plants13162254 (PMC11359158; doi:10.3390/plants13162254)
Supplement: Supplementary file 1 [file plants-13-02254-s001.zip › plants-3115576-supplementary.pdf]

Supplemental Figures:

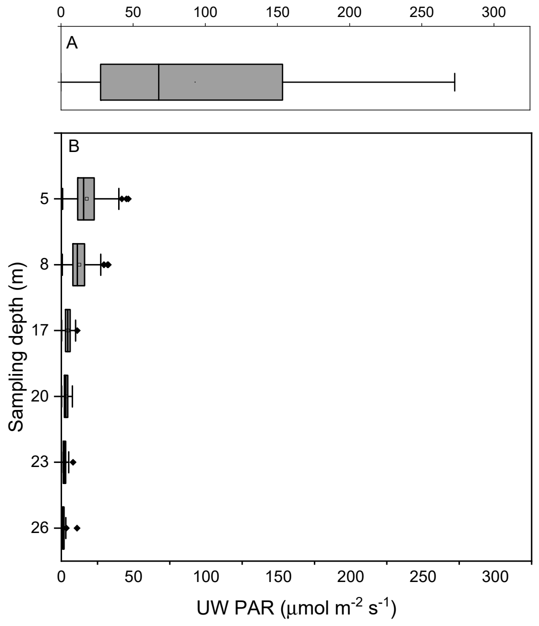

Supplemental Figure S1: Variation in underwater light availability (photosynthetically available radiation, PAR) during the growing season (Oct – Feb) of Lake Bonney (east lobe). A. moat (sampling years 2018 – 2020); B. ice-covered water column (sampling years, 1993 – 2020).

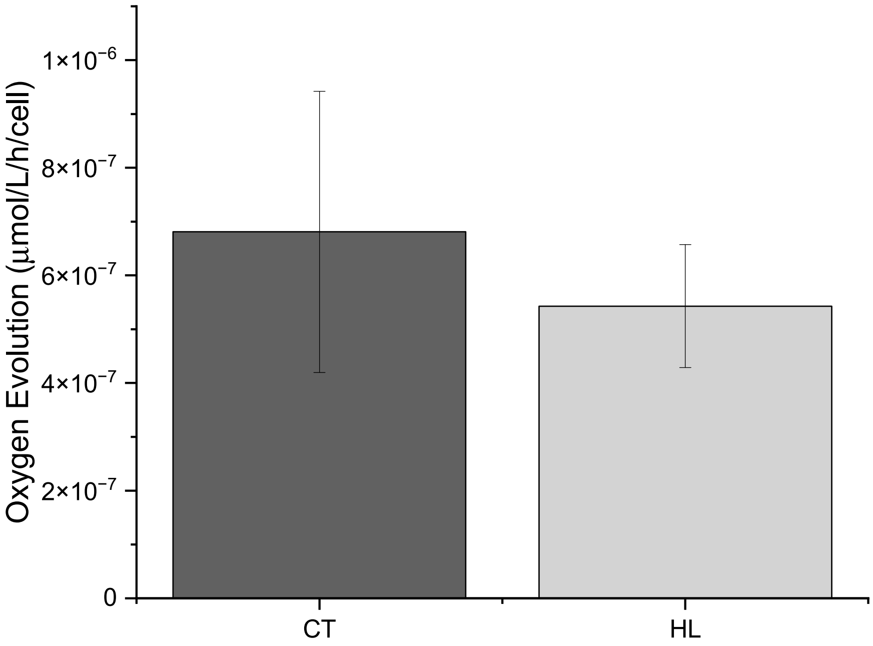

Supplemental Figure S2: Oxygen evolution rates of control and high light grown UWO241 normalized to cell counts measured using an automated cell counter (Countess II, ThermoFisher). Data are means of three replicate ±SD.
